# Emergency Department Visits for Suspected Suicide Attempts Among Persons Aged 12–25 Years Before and During the COVID-19 Pandemic — United States, January 2019–May 2021

**DOI:** 10.15585/mmwr.mm7024e1

**Published:** 2021-06-18

**Authors:** Ellen Yard, Lakshmi Radhakrishnan, Michael F. Ballesteros, Michael Sheppard, Abigail Gates, Zachary Stein, Kathleen Hartnett, Aaron Kite-Powell, Loren Rodgers, Jennifer Adjemian, Daniel C. Ehlman, Kristin Holland, Nimi Idaikkadar, Asha Ivey-Stephenson, Pedro Martinez, Royal Law, Deborah M. Stone

**Affiliations:** ^1^National Center for Injury Prevention and Control, CDC; ^2^Center for Surveillance, Epidemiology, and Laboratory Services, CDC.

Beginning in March 2020, the COVID-19 pandemic and response, which included physical distancing and stay-at-home orders, disrupted daily life in the United States. Compared with the rate in 2019, a 31% increase in the proportion of mental health–related emergency department (ED) visits occurred among adolescents aged 12–17 years in 2020 (*1*). In June 2020, 25% of surveyed adults aged 18–24 years reported experiencing suicidal ideation related to the pandemic in the past 30 days (*2*). More recent patterns of ED visits for suspected suicide attempts among these age groups are unclear. Using data from the National Syndromic Surveillance Program (NSSP),* CDC examined trends in ED visits for suspected suicide attempts^†^ during January 1, 2019–May 15, 2021, among persons aged 12–25 years, by sex, and at three distinct phases of the COVID-19 pandemic. Compared with the corresponding period in 2019, persons aged 12–25 years made fewer ED visits for suspected suicide attempts during March 29–April 25, 2020. However, by early May 2020, ED visit counts for suspected suicide attempts began increasing among adolescents aged 12–17 years, especially among girls. During July 26–August 22, 2020, the mean weekly number of ED visits for suspected suicide attempts among girls aged 12–17 years was 26.2% higher than during the same period a year earlier; during February 21–March 20, 2021, mean weekly ED visit counts for suspected suicide attempts were 50.6% higher among girls aged 12–17 years compared with the same period in 2019. Suicide prevention measures focused on young persons call for a comprehensive approach, that is adapted during times of infrastructure disruption, involving multisectoral partnerships (e.g., public health, mental health, schools, and families) and implementation of evidence-based strategies (*3*) that address the range of factors influencing suicide risk.

CDC examined NSSP ED visit data, which include approximately 71% of the nation’s EDs in 49 states (all except Hawaii) and the District of Columbia. ED visits for suspected suicide attempts were identified by using a combination of chief complaint terms and administrative discharge diagnosis codes. ED visits for suspected suicide attempts include visits for suicide attempts, as well as some nonsuicidal self-harm visits (*4*). Suspected suicide attempts were identified by querying an NSSP syndrome definition developed by CDC in partnership with state and local health departments (Supplementary Table, https://stacks.cdc.gov/view/cdc/106694). All analyses were restricted to EDs that reported consistently throughout the study period (January 1, 2019–May 15, 2021) and had at least one visit for suspected suicide attempts; 41% of those that reported consistently had one or more visits for suspected suicide attempts.^§^ Weekly counts and rates (mean number of ED visits for suspected suicide attempts/mean total number of ED visits) x 100,000) analyzed by age group (12–17 and 18–25 years) and sex were plotted across the entire study period, and analyzed for three distinct periods: spring 2020 (March 29–April 25, 2020; calendar year weeks 14–17); summer 2020 (July 26–August 22, 2020; weeks 31–34); and winter 2021 (February 21–March 20, 2021; weeks 8–11) and compared with their corresponding reference periods in 2019.^¶^ These time frames were selected as representative of distinct periods throughout the pandemic. Percent change and visit ratios (rate of ED visits for suspected suicide attempts during surveillance period/rate of ED visits for suspected suicide attempts during reference period) with 95% confidence intervals (CIs) were calculated to compare suspected suicide attempt ED visit rates by pandemic period and sex; CIs that excluded 1.0 were considered statistically significant. NSSP race and ethnicity data were not available at the national level for this analysis at the time it was conducted. All analyses were conducted using R software (version 4.0.5; R Foundation). This activity was reviewed by CDC and was conducted consistent with applicable federal law and CDC policy.**

Among adolescents aged 12–17 years, the number of weekly ED visits for suspected suicide attempts decreased during spring 2020 compared with that during 2019 (Figure 1) (Table). ED visits for suspected suicide attempts subsequently increased for both sexes. Among adolescents aged 12–17 years, mean weekly number of ED visits for suspected suicide attempts were 22.3% higher during summer 2020 and 39.1% higher during winter 2021 than during the corresponding periods in 2019, with a more pronounced increase among females. During winter 2021, ED visits for suspected suicide attempts were 50.6% higher among females compared with the same period in 2019; among males, such ED visits increased 3.7%. Among adolescents aged 12–17 years, the rate of ED visits for suspected suicide attempts also increased as the pandemic progressed (Supplementary Figure 1, https://stacks.cdc.gov/view/cdc/106695). Compared with the rate during the corresponding period in 2019, the rate of ED visits for suspected suicide attempts was 2.4 times as high during spring 2020, 1.7 times as high during summer 2020, and 2.1 times as high during winter 2021 (Table). This increase was driven largely by suspected suicide attempt visits among females.

**FIGURE 1:**
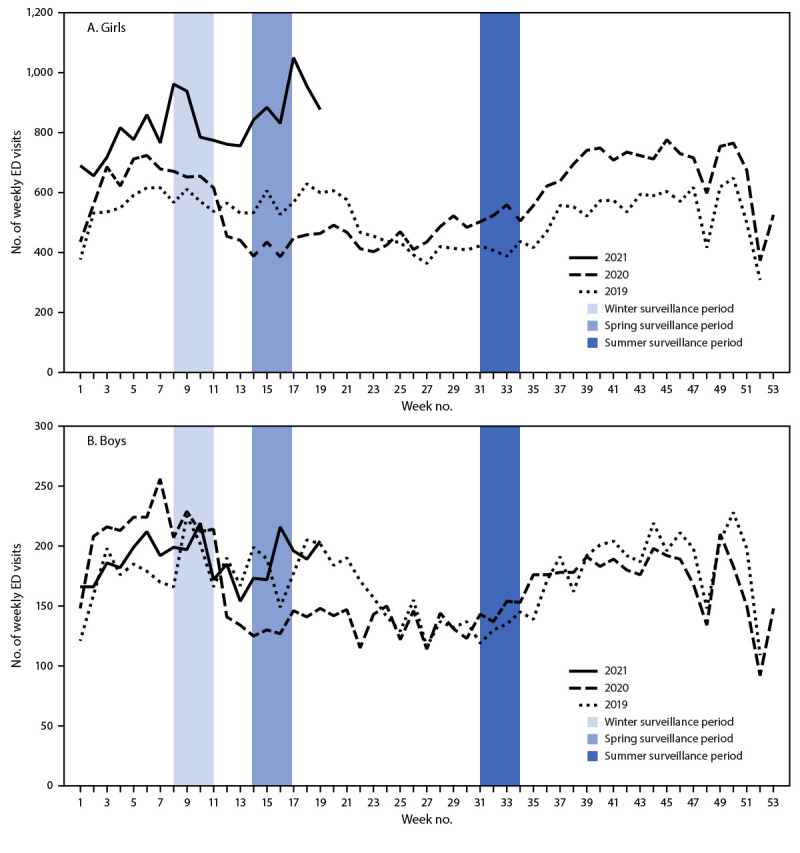
Numbers of weekly emergency department visits* for suspected suicide attempts^†^ among adolescents aged 12–17 years, by sex — National Syndromic Surveillance Program, United States, January 1, 2019–May 15, 2021 **Abbreviations:** ED = emergency department; NSSP = National Syndromic Surveillance Program. * ED visits for suspected suicide attempts were identified by querying an NSSP syndrome definition developed by CDC in partnership with state and local health departments (https://stacks.cdc.gov/view/cdc/106694). NSSP ED visit data include approximately 71% of the nation’s EDs in 49 states (all except Hawaii) and the District of Columbia. **^†^** Visits for suspected suicide attempts include visits for suicide attempts, as well as nonsuicidal self-harm.

**TABLE:** Mean weekly counts, percentage change,* visit rates,^†^ and visit ratios^§^ of emergency department visits for suspected suicide attempts^¶^ among persons aged 12–25 years — National Syndromic Surveillance Program** — United States, March 29, 2020–March 20, 2021

Surveillance period and indicators	Adolescents aged 12–17 yrs				Adults aged 18–25 yrs			
	All	Girls	Boys	Ratio^††^ for girls to boys	All	Women	Men	Ratio^††^ for women to men
**Spring 2020^§§^ weeks 14–17 (March 29–April 25)**								
Mean no. of weekly ED visits for suspected suicide attempts	540.25	408.25	131.75	N/A	646.50	385.50	257.50	N/A
% Change in mean no. of weekly ED visits for suspected suicide attempts	−26.45	−26.57	−25.56	N/A	−16.80	−20.68	−10.75	N/A
ED visit rates^†^ for suspected suicide attempts	2,750.03	3,766.75	1,499.25	N/A	815.31	827.30	789.99	N/A
Visit ratio (95% CI)	2.36 (2.23 to 2.49)	2.32 (2.17 to 2.47)	2.43 (2.17 to 2.72)	2.51 (2.28 to 2.77)	1.58 (1.50 to 1.67)	1.62 (1.51 to 1.73)	1.53 (1.41 to 1.66)	1.05 (0.97 to 1.13)
**Summer 2020: weeks 31–34 (July 26–August 22)**								
Mean no. of weekly ED visits for suspected suicide attempts	665.50	518.50	145.75	N/A	754.75	456.25	297.50	N/A
% Change in mean no. of weekly ED visits for suspected suicide attempts	22.33	26.16	10.84	N/A	−5.60	−2.82	−9.37	N/A
ED visit rates^†^ for suspected suicide attempts	1,665.09	2,360.65	812.36	N/A	588.63	589.39	587.70	N/A
Visit ratio (95% CI)	1.65 (1.56 to 1.74)	1.64 (1.54 to 1.75)	1.55 (1.38 to 1.75)	2.91 (2.65 to 3.18)	1.12 (1.06 to 1.17)	1.18 (1.10 to 1.25)	1.03 (0.95 to 1.12)	1.00 (0.93 to 1.08)
**Winter 2021: weeks 8–11 (February 21–March 20)**								
Mean no. of weekly ED visits for suspected suicide attempts	1,054.25	855.50	195.50	N/A	786.50	489.75	294.75	N/A
% Change in mean no. of weekly ED visits for suspected suicide attempts	39.13	50.55	3.71	N/A	1.68	5.83	−4.22	N/A
ED visit rates^†^ for suspected suicide attempts	2,482.32	3,600.89	1,048.00	N/A	652.98	657.15	644.35	N/A
Visit ratio (95% CI)	2.12 (2.02 to 2.22)	2.26 (2.15 to 2.39)	1.61 (1.45 to 1.77)	3.44 (3.18 to 3.71)	1.26 (1.20 to 1.33)	1.35 (1.27 to 1.44)	1.15 (1.06 to 1.24)	1.02 (0.95 to 1.10)

**Abbreviations**: CI = confidence interval; ED = emergency department; N/A = not applicable.

* Percent change in visits per week during each surveillance period was calculated as the difference in total visits between the surveillance period and the reference period, divided by the total visits during the reference period, times 100%. ([ED visits for suspected suicide attempts during surveillance period–ED visits for suspected suicide attempts during reference period]/ED visits for suspected suicide attempts during reference period*100%).

**^†^** Rate of ED visits for suspected suicide attempts = (mean number of ED visits for suspected suicide attempts/mean total number of ED visits) x 100,000.

**^§^** Visit ratios for suspected suicide attempt visits = (rate of ED visits for suspected suicide attempts during the surveillance period/rate of ED visits for suspected suicide attempts during reference period). Ratios >1 indicate a higher rate of ED visits for suspected suicide attempts during the surveillance period than during the reference period. Reference periods are as follows: for weeks 14–17, 2020 (March 29–April 25, 2020, Spring 2020): weeks 14–17, 2019 (March 21–April 27, 2019); for weeks 31–34, 2020 (July 26–August 22, 2020, Summer 2020): weeks 31–34, 2019 (July 28–August 24, 2019); for weeks 8–11, 2021 (February 21–March 20, 2021, Winter 2021): weeks 8–11, 2019 (February 17–March 16, 2019).

**^¶^** ED visits for suspected suicide attempts were defined using NSSP’s syndrome definition based on a combination of chief complaint terms and administrative discharge diagnosis codes.

** NSSP is a collaborative program among CDC, local and state health departments, and academic and private sector partners supporting the collection and analysis of electronic health data. Results in this analysis are limited to only ED encounters. As of March 31, 2021, 71% of all nonfederal EDs in the United States. (3,722) covering 49 states (all except Hawaii) and the District of Columbia contribute data to the platform daily. Of all the EDs that met the data quality criteria, 41% observed visits for suspected suicide attempts and thus were included in the analysis.

**^††^** Female to male visit ratios = (proportion of ED visits for suspected suicide attempts during surveillance period for females/proportion of ED visits for suspected suicide attempts during surveillance period for males). Ratios >1 indicate a higher proportion of suspected suicide attempt–related ED visits during the surveillance period for females compared with males.

^§§^ Data are shown only for the surveillance periods (spring 2020: March 29–April 25, 2020; summer 2020: July 26–August 22, 2020; and winter 2021: February 21–March 20, 2021). Thus, the date range is different from that in the figures, which depict the entire study period (January 1, 2019–May 15, 2021).

Among men and women aged 18–25 years, a 16.8% drop in the number of ED visits for suspected suicide attempts occurred during spring 2020 compared with the 2019 reference period (Figure 2) (Table). Although ED visits for suspected suicide attempts subsequently increased, they remained consistent with 2019 counts (Figure 2). However, the ED visit rate for suspected suicide attempts among adults aged 18–25 years was higher throughout the pandemic compared with that during 2019 (Supplementary Figure 2, https://stacks.cdc.gov/view/cdc/106696). Compared with the rate in 2019, the rate was 1.6 times as high during spring 2020, 1.1 times as high during summer 2020, and 1.3 times as high during winter 2021 (Table).

**FIGURE 2:**
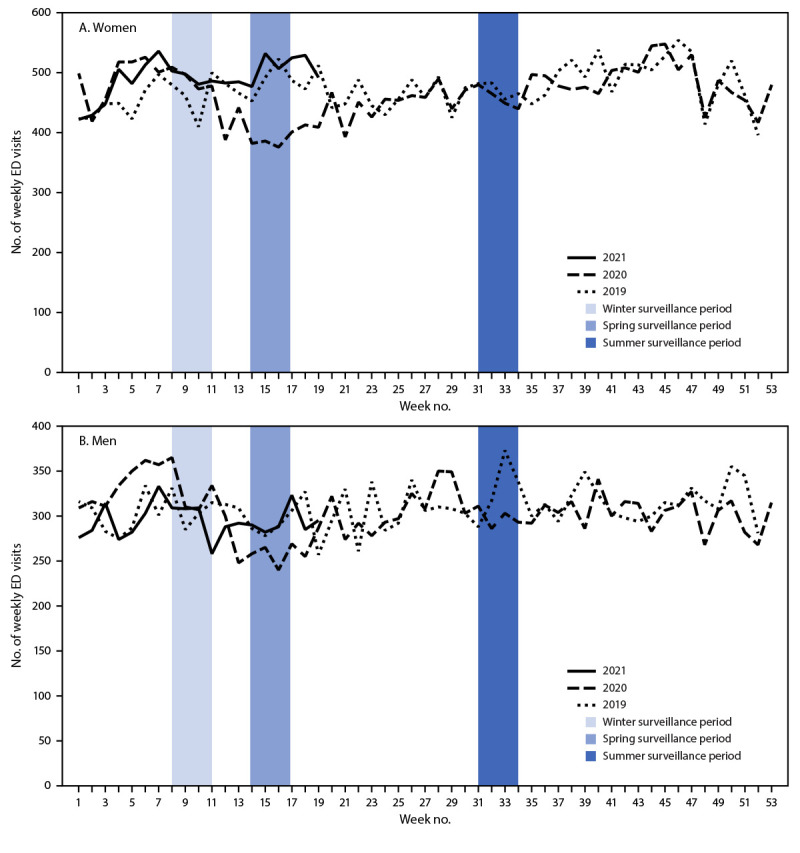
Numbers of weekly emergency department visits* for suspected suicide attempts^†^ among adults aged 18–25 years, by sex — National Syndromic Surveillance Program, United States, January 1, 2019–May 15, 2021 **Abbreviations:** ED = emergency department; NSSP = National Syndromic Surveillance Program. * ED visits for suspected suicide attempts were identified by querying an NSSP syndrome definition developed by CDC in partnership with state and local health departments (https://stacks.cdc.gov/view/cdc/106694). NSSP ED visit data include approximately 71% of the nation’s EDs in 49 states (all except Hawaii) and the District of Columbia. ^†^ Visits for suspected suicide attempts include visits for suicide attempts, as well as nonsuicidal self-harm.

## Discussion

This report expands upon previous work highlighting increases in ED visits for suspected suicide attempts earlier in the pandemic among all persons (*5*) and suggests that these trends persisted among young persons as the pandemic progressed. Compared with the corresponding period in 2019, persons aged 12–25 years made fewer ED visits for suspected suicide attempts during March 29–April 25, 2020, the period that followed the declaration of the COVID-19 pandemic as a national emergency and a concurrent 42% decrease in the total number of U.S. ED visits (*6*). However, ED visits for suspected suicide attempts increased among adolescent girls aged 12–17 years during summer 2020 and remained elevated throughout the remaining study period; the mean weekly number of these visits was 26.2% higher during summer 2020 and 50.6% higher during winter 2021 compared with the corresponding periods in 2019. The number of ED visits for suspected suicide attempts remained stable among adolescent boys aged 12–17 years and among all adults aged 18–25 years compared with the corresponding periods in 2019, although rates of ED visits for suspected suicide attempts increased.

The difference in suspected suicide attempts by sex and the increase in suspected suicide attempts among young persons, especially adolescent females, is consistent with past research: self-reported suicide attempts are consistently higher among adolescent females than among males (*7*), and research before the COVID-19 pandemic indicated that young females had both higher and increasing rates of ED visits for suicide attempts compared with males (*8*). However, the findings from this study suggest more severe distress among young females than has been identified in previous reports during the pandemic (*1*,*2*), reinforcing the need for increased attention to, and prevention for, this population. Importantly, although this report found increases in ED visits for suspected suicide attempts among adolescent females during 2020 and early 2021, this does not mean that suicide deaths have increased. Provisional mortality data found an overall decrease in the age-adjusted suicide rate from quarter 3 (July–September) of 2019 to quarter 3 of 2020. The suicide rate among young persons aged 15–24 years during this same period saw no significant change (*9*). Future analyses should further examine these provisional rates by age, sex, race, ethnicity, and geographic setting.

Some researchers have cautioned about a potential increase in suicides during the COVID-19 pandemic on account of increases in suicide risk factors; however, this study was not designed to identify the risk factors leading to increases in suspected suicide attempts (*10*). Young persons might represent a group at high risk because they might have been particularly affected by mitigation measures, such as physical distancing (including a lack of connectedness to schools, teachers, and peers); barriers to mental health treatment; increases in substance use; and anxiety about family health and economic problems, which are all risk factors for suicide. In addition, average ED visit rates for mental health concerns and suspected child abuse and neglect, risk factors for suicide attempts, also increased in 2020 compared with 2019 (*5*), potentially contributing to increases in suspected suicide attempts. Conversely, by spending more time at home together with young persons, adults might have become more aware of suicidal thoughts and behaviors, and thus been more likely to take their children to the ED.

The findings in this report are subject to at least nine limitations. First, these data are not nationally representative. Second, facility participation varies within and across states; however, data were only analyzed from facilities that reported consistently over the study period, thus minimizing the impact of reporting fluctuations on resultant trends. Third, differences in availability, coding practices, and reporting of chief complaints and discharge diagnoses from facilities might influence results returned by the syndrome definition. Fourth, distinguishing initial visits from follow-up visits for the same event was not possible, so the number of ED visits for suspected suicide attempts might be lower than presented. Fifth, NSSP race and ethnicity data were not available at the national level for this analysis at the time it was conducted, so analyses of differences among racial/ethnic groups was not possible. Sixth, these data likely underrepresent the true prevalence of suspected suicide attempts because persons with less severe injuries might be less likely to seek emergency care during the pandemic when many persons avoided medical settings to reduce the risk for contracting COVID-19. Seventh, the suspected suicide attempt syndrome definition excludes some, but not all, visits for nonsuicidal self-harm. Eighth, the sharp decline in all ED visits during the pandemic likely affected the number and proportion of visits for suspected suicide attempts (*6*). Finally, this analysis was not designed to determine whether a causal link existed between these trends and the COVID-19 pandemic.

 Suicide can be prevented through a comprehensive approach that supports persons from becoming suicidal as well as persons who are at increased risk for suicide.^††^ Such an approach involves multisectoral partnerships (e.g., public health, mental health, schools, and families) and implementation of evidence-based strategies to address the range of factors influencing suicide attempts, which is a leading risk factor for suicide (*3*). Strategies specific to young persons include preventing and mitigating adverse childhood experiences, strengthening economic supports for families, limiting access to lethal means (e.g., safe storage of medications and firearms), training community and school staff members and others to learn the signs of suicide risk and how to respond, improving access and delivery of evidence-based care, increasing young persons’ social connectedness and coping skills, and following safe messaging by the media and in schools after a suicide (*3*). Widely implementing these comprehensive prevention strategies across the United States, including adapting these strategies during times of infrastructure disruption, such as during the pandemic, can contribute to healthy development and prevent suicide among young persons.

**Summary**

**What is already known about this topic?**

During 2020, the proportion of mental health–related emergency department (ED) visits among adolescents aged 12–17 years increased 31% compared with that during 2019.

**What is added by this report?**

In May 2020, during the COVID-19 pandemic, ED visits for suspected suicide attempts began to increase among adolescents aged 12–17 years, especially girls. During February 21–March 20, 2021, suspected suicide attempt ED visits were 50.6% higher among girls aged 12–17 years than during the same period in 2019; among boys aged 12–17 years, suspected suicide attempt ED visits increased 3.7%.

**What are the implications for public health practice?**

Suicide prevention requires a comprehensive approach that is adapted during times of infrastructure disruption, involves multisectoral partnerships and implements evidence-based strategies to address the range of factors influencing suicide risk.

## Footnotes

* NSSP is a collaborative program among CDC, federal partners, local and state health departments, and academic and private sector partners to support the collection and analysis of electronic health data from EDs, urgent and ambulatory care centers, inpatient health care facilities, and laboratories.

† Analysis was limited to ED encounters. As of March 31, 2021, a total of 3,722 EDs covering 49 states (all except Hawaii) and the District of Columbia contributed data to the platform daily, including data from 71% of all nonfederal EDs in the United States.

§ To limit the impact of data quality on trends, all analyses were restricted to facilities with a coefficient of variation <30 throughout the analysis period January 2019–May 2021 so that only consistently reporting facilities were included. Of all the EDs that met the data quality criteria, 41% had visits and thus were included in the analysis.

¶ Percent change in visits per week during each surveillance period was calculated as the difference in total visits between the surveillance period and the reference period, divided by the total visits during the reference period, times 100%. ([ED visits for suspected suicide attempts during surveillance period–ED visits for suspected suicide attempts during reference period]/ED visits for suspected suicide attempts during reference period*100%).

** 45 C.F.R. part 46, 21 C.F.R. part 56; 42 U.S.C. Sect. 241(d); 5 U.S.C. Sect. 552a; 44 U.S.C. Sect. 3501 et seq.

††
https://www.cdc.gov/suicide/programs/csp/index.html
